# Replicating Cortical Signatures May Open the Possibility for “Transplanting” Brain States via Brain Entrainment

**DOI:** 10.3389/fnhum.2021.710003

**Published:** 2021-09-22

**Authors:** Alexander Poltorak

**Affiliations:** ^1^Neuroenhancement Lab, Suffern, NY, United States; ^2^The City College of New York, New York, NY, United States

**Keywords:** mental state, brain wave, transcranial stimulation, sensory stimulation, photobiomodulation, brain state, brain entrainment, cortical signature

## Abstract

Brain states, which correlate with specific motor, cognitive, and emotional states, may be monitored with noninvasive techniques such as electroencephalography (EEG) and magnetoencephalography (MEG) that measure macroscopic cortical activity manifested as oscillatory network dynamics. These rhythmic cortical signatures provide insight into the neuronal activity used to identify pathological cortical function in numerous neurological and psychiatric conditions. Sensory and transcranial stimulation, entraining the brain with specific brain rhythms, can effectively induce desired brain states (such as state of sleep or state of attention) correlated with such cortical rhythms. Because brain states have distinct neural correlates, it may be possible to induce a desired brain state by replicating these neural correlates through stimulation. To do so, we propose recording brain waves from a “donor” in a particular brain state using EEG/MEG to extract cortical signatures of the brain state. These cortical signatures would then be inverted and used to entrain the brain of a “recipient” *via* sensory or transcranial stimulation. We propose that brain states may thus be transferred between people by acquiring an associated cortical signature from a donor, which, following processing, may be applied to a recipient through sensory or transcranial stimulation. This technique may provide a novel and effective neuromodulation approach to the noninvasive, non-pharmacological treatment of a variety of psychiatric and neurological disorders for which current treatments are mostly limited to pharmacotherapeutic interventions.

## Introduction

It has been suggested (Crick and Koch, 1990; see also Rees et al., 2002; Crick and Koch, 2006) that every mental state is expressed through unique neural signals, such as neuronal oscillations, that are correlated with mood, cognition, and motor functions. Crick and Koch (1990) thus proposed that neuronal oscillations are the neural correlates of mental states. However, individual neuronal oscillations are difficult to observe, since doing so usually requires inserting electrodes into the brain. The oscillations of neurons and groups of neurons produce macroscopic activity signatures through the summation of synchronized electromagnetic signals from individual neurons (often referred to as brain rhythms or brain waves), which can be observed using noninvasive techniques such as electroencephalography (EEG) or magnetoencephalography (MEG).^1^ We posit that these brain waves can be considered neural correlates of brain states. We hypothesize that it may be possible to induce a desired brain state by replicating its neural correlates through sensory or transcranial stimulation.

## Brain States^2^

Brain states refer to the synthesis of endogenous activity in part shaped by previous experience and current sensory input to create an overall state accessible to objective measurement. A brain state is sometimes described as a snapshot in time of the central nervous system (CNS). However, such a static picture taken in a particular moment in time does not reflect temporal patterns that are critical for describing the brain state. Brain states are expressed as patterns of active neurons, active synapses, and neural oscillations. When speaking of brain states, we are concerned with measurable patterns of neural activity and the ability to actively manipulate these spatiotemporal patterns in order to alter behavior. Some define brain states as patterns of synchronous neural firing (Brown, 2006). We define brain states here as macroscopic patterns of electrical activity in the brain that repeatedly occur as a function of endogenous neuronal dynamics and their response to sensory input. The neural correlates of brain states are rhythmic activity patterns resulting from neuronal oscillations. These rhythmic activity patterns represent measurable cortical signatures.

Brain states include conscious and unconscious states. Conscious states include *inter alia* the state of focus, the state of flow, and various emotional, motor, and cognitive states as well as altered states of consciousness. Unconscious states include various stages of sleep and the state of general anesthesia (Gervasoni et al., 2004).^3^ Sleep states are of particular interest, because each stage of sleep is (at least partially) defined by prominent rhythmic network activity in specific frequency bands (Steriade, 2006). The first non–rapid eye movement (non-REM) stage of sleep, N1, is referred to as relaxed wakefulness. During this transitional stage the alpha waves (8–13 Hz) are replaced with the theta waves (5–7 Hz). The second non-REM stage of sleep, N2, is characterized by the appearance of sleep spindles [short bursts of high-frequency neural activity further subdivided into fast spindles of 11–13 Hz and slow spindles of 13–15 Hz (Cline, 2011)] and K-complexes (single long delta waves that last for only a second). The third non-REM stage of sleep, N3,^4^ is deep sleep, during which theta waves are replaced with delta waves (4 Hz and below) (see also Lin et al., 2020). It is worth pointing out that classification of sleep stages is not based exclusively on the detection of a single, dominant frequency but rather accounts for a series of criteria about frequency, amplitude, waveform shape, the occurrence of transient synchronized events (such as spindles and K-complexes), auxiliary signals about muscle tone and eye movements, and so forth.

The complexity of neural activity suggests that the reduction of brain states to an oscillatory signal of a single frequency can be expected to fail to capture the full complexity of brain states. Indeed, the conventional focus on individual oscillations and frequency bands as signatures of brain states is limiting. Nevertheless, the research literature reveals an almost exclusive focus on single-frequency stimulation waveforms for the modulation of brain states with noninvasive brain stimulation (such as the use of a sine wave in transcranial alternating current stimulation, tACS). We hypothesize that taking into account the rich temporal structure of brain activity beyond a single (dominant) frequency in the spectrum should enable more effective brain entrainment through stimulation. Thus, a naturalistic modulation waveform based on cortical signatures extracted from endogenous brain waves of a sleeping subject contains all attendant rhythms, which dynamically change in their prominence as the subject moves through various stages of the healthy sleep cycle. Using such a naturalistic, multifrequency dynamic waveform for stimulation is expected to be more efficacious than using a single static frequency, typically 5 Hz, that is traditionally used to induce sleep. Thus, neuromodulation with such naturalistic waveforms may prove beneficial in treating insomnia and other sleep disorders (Gebodh et al., 2019, 2020).

## Brain Waves as Neural Correlates of Brain States

Oscillating neurons produce emergent meso- and macroscale rhythmic electric signals often referred to as brain rhythms or brain waves, which can be detected and recorded using noninvasive techniques. Can we consider brain waves the neural correlates of brain states?

To be sure, brain waves are not identical with brain states and may not contain all information encoded in brain states. Measurements of brain waves, of course, are subject to all the limitations (such as noise or signal averaging across vast numbers of neurons) of the instruments being utilized, and loss of information is inevitable. However, we posit a bilateral correlation between brain waves and brain states. There is no doubt that brain states cause (*via* neuronal oscillations) brain waves. The question is, Do brain waves cause brain states? We hypothesize that specific spatiotemporal patterns of neural activity in the brain that are correlated with particular brain states can also cause these brain states. So, if brain state A causes brain waves X (characterized by their frequencies and spatial distribution), replicating the same brain waves X (at least in the same person) results in brain state A. A growing number of studies have recently shown that modulating brain rhythms causes changes in cognitive performance, indicative of a causal role of brain oscillations in brain states (Grover et al., 2021; Romei et al., 2016; Fröhlich et al., 2015). Sensory entrainment was shown to result in behavioral (perceptual) changes (Mathewson et al., 2010). Therefore, we posit a causal relationship that can be experimentally investigated. This conjecture is more likely to hold true for brain states that are predominantly cortical states, because cortical brain states are more likely to be the product of cortical activity patterns such that, when a specific cortical activity is induced through stimulation, the brain switches to the corresponding state.

On the other hand, this conjecture is less likely to hold for brain states that involve deep brain structures and specialized nuclei or the brain stem. For example, it can be imagined that certain spatiotemporal patterns are indicative of an underlying brain state not accessible with noninvasive measurements of brain activity. Imposing the corresponding activity patterns may fail to induce the corresponding brain state, since controlling (e.g., stimulating) inaccessible subcortical brain areas (or, more broader, physiological processes) would be necessary. The state of sleep, for example, is regulated by the hypothalamus [including suprachiasmatic nuclei (SCN) responsible for circadian rhythms or the ventrolateral preoptic nucleus (VLPO)], brain stem, thalamus, pineal gland, basal forebrain, and amygdala. There is little reason to expect that the cortical signatures of the sleeping donor would entrain subcortical structures (such as the SCN or VLPO) of the recipient. Nevertheless, recent research confirms that cortical structures play a role in regulating sleep homeostasis and global sleep-wake dynamics (Krone et al., 2021), leaving room for the possibility that the state of sleep may be induced by entrainment, as suggested here.

This distinction results from the fact that neither recording nor stimulation techniques have access to all physiological processes that (at least in theory) contribute to a brain state.

Functional neuroimaging such as EEG or MEG can capture the neuronal activity of localized brain regions, which correlate with distinct cognitive or behavioral states (brain states). EEG recordings have demonstrated, for example, that the pattern of brain activity changes during meditative acts, and frontal cortex EEG activity has been associated with emotion induction and regulation (Dennis and Solomon, 2010; Yu et al., 2011). EEG recordings reflect ionic fluctuations resulting from neuronal communication in the cortex arising from dendritic depolarizations (Nunez and Srinivasan, 2006). Alternatively, MEG measurements reflect intracellular ionic fluctuations resulting from action potentials (Hämäläinen et al., 1993). In both cases, output measures correlate with localized cortical activity.^5^

Electroencephalography or MEG recordings can be used to extract cortical signatures. This can be done using statistical techniques such as principal components analysis (CRI), independent components analysis (ICA), spectral analysis and similar techniques, or machine-learning pattern recognition.

## Neuromodulation

These cortical signatures may be inverted in order to stimulate cortical activity. Endogenous electric fields (i.e., brain waves) directly entrain neuronal network activity (Fröhlich and McCormick, 2010). This fundamental discovery enables the design of low-amplitude electromagnetic brain stimulation modalities to modulate and enhance oscillatory network dynamics. Indeed, transcranial electric stimulation (TES; Annarumma et al., 2018), including tACS (Antal et al., 2008) and transcranial direct current stimulation (tDCS; Nitsche, 2000; see also Utz et al., 2010), is used to electrically stimulate cortical activity, whereas transcranial magnetic stimulation (TMS; Barker, 1985; McLean, 2019) uses a spatially targeted magnetic field to achieve a similar endpoint of controlling electric activity in brain networks. Typical brain stimulation methods use constant stimulation as a waveform (e.g., tDCS) or a synthetic waveform, which may be a step function modulated on a direct current, such as “electrosleep” (Robinovitch, 1911), a sinusoid modulated on an oscillatory direct current (osc-tDCS; D’Atri et al., 2016), or a single fixed frequency of alternating current (tACS) (Rosa and Lisanby, 2012; Herrmann, 2013). Helfrich et al. (2014) utilized simultaneous tACS stimulation combined with EEG recordings to show that, when tACS was applied to the parieto-occipital lobe of the brain, alpha oscillations increased and became synchronized with the entrainment frequency. Similarly, brain entrainment can be achieved by stimulating the brain *via* sensory pathways using visual or auditory stimuli (Notbohm et al., 2016).

Whereas TES and TMS modulate neuronal activity, entrainment using sensory pathways can also change the brain state. Indeed, the endogenous circadian rhythm responsible for the transition from the sleeping state to the waking state is primarily entrained by light (Bedrosian et al., 2013).

Endogenous circadian rhythms are entrained – both in terms of the firing of action potentials and gene expression – to the diurnal cycle. Although such entrainment happens on a distinctly slower timescale than the neuronal oscillations discussed here so far, this serves as an example of how sensory input alters rhythmic processes in the brain. Overall, we emphasize that rhythmic brain activity is a reflection of rhythmic processes in our environment (from the slow timescales of the day-night cycle to the fast oscillations in auditory input) across an extremely wide band of frequencies.

It is noteworthy that SCN owe their ability to sense the cycle of light and darkness to the retinohypothalamic tract (RHT), connecting the intrinsically photosensitive retinal ganglion cells (ipRGC) in the retina to SCN *via* the optic nerve (Gooley et al., 2001). The RHT provides a natural pathway to use photobiomodulation for stimulating the hypothalamus and, through it, other structures of the brain.

The brain is very efficient at processing light and sound stimuli. Therefore, stimulation with light (photobiomodulation) or sound (isochronic tones or binaural beats) holds significant promise for entraining a brain state. These natural stimuli have a greater likelihood of reproducing naturalistic brain rhythms than single frequency stimulation waveforms do [see, for example, Pérez et al. (2017)].

We hypothesize that sensory stimulation is particularly promising since it uses established neuronal circuits for the brain to respond to external input and avoids the pitfalls inherent in recent debates about the amount of energy actually delivered to the brain by transcranial current stimulation. However, it is also conceivable that certain structural and functional brain networks are configured to be robust to perturbations caused by sensory input. These circuits may be preferentially modulated by electromagnetic stimulation. More research is needed to establish the specific utility of sensory vs. electromagnetic stimulation as a function of the specific scientific and clinical application.

Here, we propose using these techniques to transplant a desirable brain state from one subject (the “donor”), who is in the desired brain state, to another subject (the “recipient,” who is either another person or the same subject at another time), who wishes to attain this brain state. We propose to do that by recording and subsequently inducing desired brain states. Thus, EEG or MEG may be used to record the brain state of the first subject (the donor), from which cortical signatures may be extracted and inverted to create modulation waveforms. Such waveforms may then be modulated on various physical signals, such as direct or alternating current, magnetic field, light, sound, or vibration and applied *via* TES (tDCS, osc-tDCS, and tACS), TMS, or sensory (visual, auditory, or tactile) stimulation to entrain the brain of the recipient with the cortical signatures of the donor, thereby inducing the cognitive-behavioral state of the donor within the recipient.

In a pilot study, we investigated this technique (transplanting brain state) in the domain of sleep (Gebodh et al., 2019, 2020). In these studies, we used TES (tACS modulated with endogenous sleep-derived waveform, “tESD”). For the reasons explained above, light and sound appear to be more promising modalities for inducing the desired brain state than direct brain stimulation with TES would be.

Generally, we propose to investigate the notion of “transplanting” brain states, including sleep, attention, and learning as well as emotional valence. Attention states in the brain primarily result from the cognitive control process of the selective direction of information processing resources to behaviorally relevant stimuli and from active suppression of the detection and processing of irrelevant stimuli (Gulbinaite et al., 2017). Thus, functionally, attention serves as both a filter, eliminating less relevant stimuli from conscious perception, and an amplifier, increasing the salience of behaviorally relevant stimuli. This cognitive state is associated with specific neuronal oscillations (Schroeder et al., 2010), which may be captured by EEG or MEG. The neural oscillations associated with attention have been shown to be disrupted in a number of conditions, including epilepsy (Besle et al., 2011), dyslexia (Thomson and Goswami, 2008; Soltész et al., 2013; Leong and Goswami, 2014), and schizophrenia (Lakatos et al., 2013). Therefore, acquiring a brain wave signature during states of attention in a healthy “donor” may prove valuable when applied to a recipient exhibiting attention deficits associated with disrupted or otherwise irregular cortical oscillations.

Stimulating the brain using a waveform with a fixed frequency that significantly differs from the frequency of the endogenous brain waves (regular or irregular), as frequently done in TES and TMS studies, may cause interference and is unlikely to change the rhythm of endogenous brain waves. In contrast, brain entrainment should optimally start where the brain is, not where it is desired to be. The Arnold tongue is a theoretical framework for entrainment, essentially suggesting that it is easier to entrain oscillations closer to endogenous frequencies within a given subject. Thus, neuromodulation with a dynamic waveform used for entrainment should start at the current frequency of the endogenous brain waves since such close matching of stimulation and endogenous frequency is required for phase locking as indicated by the Arnold tongue (Ali, 2013) for entrainment of neural oscillations. Once entrainment is achieved at this initial frequency, the frequency of stimulation can be gradually changed to move the endogenous rhythm toward the desired frequency in order to achieve successful entrainment at the desired target frequency (Thut et al., 2011; Notbohm et al., 2016). This approach also avoids interference issues.

Previous research shows that memory functions are acutely sensitive to neural entrainment and may be disrupted *via* TMS (Hanslmayr et al., 2014), indicating the possibility of an inverse, positive entrainment of these oscillations.

Similarly, emotional arousal and valence are correlated with distinct cortical signatures observable through EEG (Allen et al., 2018). Previous data indicate that happiness resulting from musical experience, for instance, is associated with increased theta frequency oscillations in the left frontal cortical hemisphere (Rogenmoser et al., 2016). Cortical oscillations associated with negative affect conversely correlate with decreased theta frequency oscillations in this same region. Notably, aberrant cortical oscillations have been observed in a range of affective disorders, including major depression (Van der Vinne et al., 2017). Indeed, the left frontal hemisphere exhibits disrupted cortical rhythms in patients diagnosed with major depression when compared with healthy controls (Nusslock et al., 2018). Similar data have highlighted cortical asymmetry of frontal lobe oscillations in post-traumatic stress disorder (PTSD; Meyer et al., 2018). Simple cortical entrainment *via* binaural beat stimulation has already proven adequate for inducing specific emotional states (Chaieb et al., 2015). More directly, cranial electrotherapy has been demonstrated as an effective treatment for depression, anxiety, and certain forms of insomnia (Kirsch and Nichols, 2013). In fact, certain forms of depression may respond better to transcranial approaches, such as TMS, as has been demonstrated in early data on patients with treatment-resistant major depression (George, 2000; Rosenberg et al., 2010).

## Transplanting Brain States

Thus, our proposed approach to “transplanting” (transferring) brain states by replicating neural correlates of the donor’s state in a recipient (who may be a different person or the same person at a different time) is founded on two primary principles. First, we posit that cortical signatures found in brain waves are neural correlates of brain states. A large body of literature has identified distinct, measurable cortical signatures associated with specific brain states, ranging from those defining the sleep/wake cycle to those underlying emotional experiences. Second, TES, TMS, and sensory stimulation by light and sound have been repeatedly demonstrated as efficacious, safe means by which cortical rhythms may be entrained with a high degree of location-specificity, with sensory stimulation using light or sound holding particular promise for brain entrainment. Third, we posit that brain waves resulting from brain entrainment causally induce the desired brain state associated with the cortical signatures that are encoded in the modulation waveforms used for entrainment. Thus, we assume a bidirectional causal relationship between brain states and cortical signatures found in brain waves. The combination of a mounting body of literature, as cited in this paper, supports this conjecture.

To be sure, brains differ. While the donor and the recipient may on occasion be the same person, when the donor and the recipient are not the same individual, they can be expected to differ in skull structure, brain size, and brain morphology. Moreover, the phase lag between oscillations and stimulus can differ across individuals during neural entrainment. The same is true for the optimal timing of tACS when it is applied to modulate entrainment. Although it is possible to adjust the waveform based on the specifics of the recipient brain obtained by fMRI and using computational models of the brain, this problem can be sidestepped entirely by using sensory stimulation that does not act on the brain itself but rather acts on the sensory organs and allows the brain to assimilate the signals it receives from these sensory organs on its own terms. This is yet another reason why sensory stimulation using visual and/or auditory pathways seems highly preferable to using TES or TMS.

The complexity of identifying and transplanting brain states should not be underestimated. We can investigate the brain on microscopic neurochemical levels or as a complex and widely distributed network. It is not immediately obvious which is the correct level to be acquiring and transplanting patterns that would affect changes in behavior (that is, mental states). For example, the macroscopic representations measured by EEG may be sufficient to transplant generic physiological states, such as a state associated with a specific sleep stage. However, more refined states, such as specific cognitive states, may require a higher spatial resolution to be fully captured and transplanted by noninvasive methods. Cortical signatures and representation patterns have to be carefully investigated before transplanting or replicating subjective contents of cognitive processes. More research is needed to better understand the representation patterns of specific cognitive states.

It is also possible that cortical activity signals as measured by EEG do not fully capture certain brain states. For example, rapid eye movement (REM) sleep can be distinguished from waking activity by the absence of muscle tone in major muscle groups as determined by electromyography (EMG). Therefore, additional physiological signals can be used to capture brain states more fully. In particular, capturing the status of the autonomic nervous system, such as, for example, the dynamic balance of sympathetic and parasympathetic activity reflected in measures such as heart rate variability, may turn out to be necessary for high-fidelity identification of brain states. These signals are also open to noninvasive modulation, such as through the stimulation of the vagus nerve.^6^

Finally, it is worth discussing the difference between online and offline effects of stimulation. The current brain stimulation literature suggests that cortical states can be effectively enhanced during stimulation, what are referred to as “online” effects. The mechanism by which more long-lasting effects occur in brain networks after the conclusion of stimulation remains an unanswered question. Transplanting brain states does not hinge on unraveling these mechanisms, since it focuses on augmenting, restoring, and inducing brain states with stimulation. Brief perturbations may be sufficient to switch to another state either with or without continued stimulation. The hypotheses discussed herein are compatible with both approaches. In either approach, the main mechanisms are based on the fundamental property of neuronal oscillations to respond to (weak) perturbations through entrainment.

## Conclusion

Together these findings provide the basis for our hypothesis that brain states can be “transplanted” (transferred) and provide the means by which a cortical signature may be obtained *via* EEG or EMG associated with the desired brain state of a “donor” that may, in turn, be processed, inverted, and subsequently applied to a “recipient” – who may be another person, or the same person at another time – to induce this state through cortical rhythm entrainment using preferably sensory stimuli, such as light or sound, a combination thereof, or, possibly, TES or TMS. Theoretical considerations suggest that this hypothesis is plausible and deserves experimental verification. Importantly, using cortical signatures acquired from a donor, rather than a fixed-frequency synthetic waveform stimulation as is currently typical for TES techniques, offers the distinct advantage of replicating multiphasic, multifrequency, temporally dynamic, naturalistic signals, which we believe are more likely to modulate neuronal network activity effectively (Fröhlich and McCormick, 2010) and, more important, induce naturalistic brain states due to the additional information contained in the complete spectrum of macroscale brain signals. Therefore, this technique may provide a novel and effective neuromodulation approach to the noninvasive, non-pharmacological treatment of a variety of psychiatric and neurological disorders for which current treatments are mostly limited to pharmacotherapeutic interventions.

## Data Availability Statement

The original contributions presented in the study are included in the article/Supplementary Material, further inquiries can be directed to the corresponding author/s.

## Author Contributions

The author confirms being the sole contributor of this work and has approved it for publication.

## Conflict of Interest

The author is the President of Neuroenhancement Lab, Inc., and has several patent applications pending for inventions related to the subject of this article.

## Publisher’s Note

All claims expressed in this article are solely those of the authors and do not necessarily represent those of their affiliated organizations, or those of the publisher, the editors and the reviewers. Any product that may be evaluated in this article, or claim that may be made by its manufacturer, is not guaranteed or endorsed by the publisher.

## Footnotes

1 Electroencephalography measures electrical activity with respect to a reference point and does not provide strong spatial localization of the signal. MEG measures the magnetic fields generated by electrical activity and provides better spatial location. However, MEG is biased toward measuring activity that originates in the sulci and activity close to the surface, since magnetic field activity degrades more quickly than electrical activity does.

2 In the original version of this paper (Poltorak, 2019), we referred to mental states. The term “mental states,” however, is ambiguous and ill defined. There is no consensus about what mental states are and the nature of their relationship with the brain. According to identity theory, mental states are identical with brain states (Payne, 2021). Others disagree (Chalmers, 1995). While this is a very important question in the philosophy of mind, it is not the subject of the present inquiry. To avoid confusion, henceforth we refer to brain states. Our theory concerns brain states as defined by measurable patterns of neural activity.

3 An example of a state that is not a pure brain state is a state of relaxation, which is predominantly a state of the peripheral nervous system (PNS), not the CNS. The PNS regulates the relaxation state, which includes relaxation of the skeletal muscles regulated by the somatic nervous system, relaxation of smooth muscles as well as vasodilation, the slowing of the heartbeat and respiration, etc., all regulated by the autonomous nervous system (ANS). To induce this state, one must stimulate the vagus nerve, not the brain. Indeed, it is the excitation of the parasympathetic nervous system that causes the state of relaxation.

4 Previously, N3 was subdivided into N3 and N4. For a summary of the four-stage sleep classification, see for example (Purves et al., 2001).

5 Electroencephalography signals are not easy to localize. However, the overall macroscopic EEG signal reflects smaller-scale endogenous rhythmic processes.

6 Combining vagus nerve stimulation (VNS) with brain entrainment is a promising avenue for investigation, but one must be cautious of possible interference. It has been shown that VNS significantly increased and decorrelated spontaneous activity and suppressed entrainment at 6–8 Hz (Nichols et al., 2011).
